# Modulation of the microbiota across different intestinal segments by Rifaximin in PI-IBS mice

**DOI:** 10.1186/s12866-023-02772-6

**Published:** 2023-01-19

**Authors:** Shengyan Zhang, Gaichao Hong, Gangping Li, Wei Qian, Yu Jin, Xiaohua Hou

**Affiliations:** Division of Gastroenterology, Union Hospital, Tongji Medical College, Huazhong University of Science and Technology, Wuhan, 430022 China

**Keywords:** PI-IBS, Rifaximin, Gut microbiota

## Abstract

**Background:**

Rifaximin has been increasingly applied in irritable bowel syndrome (IBS) treatment. Whether there were differences in the effects of rifaximin on microbiota from different intestinal segments, especially the small intestine where rifaximin predominantly acted, has not been confirmed.

**Methods:**

In this study, we used *Trichinella spiralis* infection to induce post infectious irritable bowel syndrome (PI-IBS) and measured visceral sensitivity of mice by means of abdominal withdrawal reflex (AWR) tests to colorectal distention (CRD). We compared the effects of rifaximin on the composition of ileal, colonic mucosal and fecal microbiota in PI-IBS mice.

**Results:**

Rifaximin significantly reduced AWR scores and increased pain threshold in PI-IBS mice, and this effect was associated with the change in the relative abundance of ileal mucosal microbiota. Rifaximin could obviously decrease ileum mucosal microbiota alpha diversity assessed by Shannon microbial diversity index. Meanwhile, the analysis of beta diversity and relative abundance of microbiota at phylum, family and genus levels showed that rifaximin could improve the microbiota structure of ileal mucosa. However, for colonic mucosal and fecal microbiota, this effect of rifaximin was not obvious. Rifaximin could reshape the correlation of genera between different intestinal segments.

**Conclusion:**

Rifaximin improved visceral hypersensitivity in PI-IBS mice. Rifaximin mainly affected ileal mucosal microbiota, and its improvement effect on IBS might be closely related to the improvement of ileal microbiota structure.

**Supplementary Information:**

The online version contains supplementary material available at 10.1186/s12866-023-02772-6.

## Introduction

IBS is a chronic gastrointestinal disease characterized by recurrent abdominal pain and change in defecation habits [1]. In recent years, studies have found that, in addition to psychological factors, gastrointestinal pathological changes such as small intestinal bacterial overgrowth (SIBO), gut dysbiosis, history of gastrointestinal infection also played an important role in the occurrence and development of IBS symptoms [2–4]. Therefore, More and more studies have paid attention to gut microbiota in IBS [5–8].

Rifaximin is a semi-synthetic rifamycin drug [9]. Compared with rifamycin, it has an additional pyridoimidazole ring, which makes it difficult to be absorbed [10]. So the effect of rifaximin is limited to the gastrointestinal tract. Moreover, its high bile solubility makes it predominantly function in small intestine. In colon, where there is plenty of water, rifaximin’s drug availability and efficacy are extremely limited [9, 11]. In vivo the metabolism of rifaximin is relatively simple, for it do not show any drug-drug interactions [12], and is excreted in the feces as unchanged drug [13]. Rifaximin shows broad-spectrum antibacterial activity against aerobic and anaerobic bacteria, including Gram-positive and Gram-negative bacteria by binding to the β-subunit of bacterial DNA-dependent RNA polymerase, which is important for bacterial RNA synthesis [14]. In recent years, the efficacy of rifaximin in IBS has been fully affirmed. Study has shown that rifaximin can effectively relieve symptoms such as bloating, abdominal pain, and loose or watery stools among IBS patients who did not suffer constipation [15],and it was approved for the treatment for IBS-D by FDA in 2015 [16, 17]. The regulation of microbiota structure as a possible mechanism of rifaximin has been recognized widely [16, 18]. However, most studies focus on the effect of rifaximin on fecal microbiota and neglected whether and how rifaximin affected microbiota in different parts of the intestine, including the small intestine. In addition, there are large differences between mucosal and luminal (fecal) microbial composition [19]. In view of the unique pharmacokinetic characteristics of rifaximin and the close relationship between mucosal-associated microbiota and host epithelial, immune cells and the enteric nervous system, it is particularly important to explore the influence of rifaximin on mucosal microbiota from different intestinal segments.

A large number of studies have confirmed that *Trichinella spiralis* infected mice could be used as an animal model to comprehensively simulate the clinical characteristics of PI-IBS [20–22].In this study, we used this model to describe the changes of microbiota composition in ileal mucosa, colonic mucosa and feces after rifaximin treatment. We found that rifaximin had different effects on different intestinal segments. Compared with colonic mucosa and feces, rifaximin had the most significant effect on ileal mucosa microbiota, and it could improve ileal microbial dysbiosis in PI-IBS mice and restore them to a similar microbiota structure to normal mice.

## Materials and methods

### Establishment of the mouse model of PI-IBS

Male NIH Swiss mice (6–8 weeks old, No. SCXK2008-0002), obtained from Medical Animal Laboratory center of Guangdong, were used in this study. Mice were housed under specific pathogen-free conditions in the animal facility of Tongji Medical College, Wuhan, China. Mice were fed with autoclaved rodent diet and sterile water, and kept at a constant temperature (22–23 °C). All the animals were kept and used for the experiment in accordance with the ethics and regulation of procedures approved by the Ethics Committee of Animal Experimentation, Tongji Medical College. *T. spriralis* was graciously provide by the department of Parasitology at Huazhong University of Science and Technology. A previously described technique for obtaining the larvae was used [23]. Mice were randomly divided into three group, namely the control group, PI-IBS group and Rifaximin group. Mice of PI-IBS group and Rifaximin group were infected via oral gavage with 350–400 T*. spiralis* larvae in 0.2 ml phosphate-buffered saline (PBS). Control mice were gavaged with 0.2 ml PBS only. PI-IBS model was established after 8 weeks. Then according to the results of the pre-experiment, mice of Rifaximin group were intragastrically administered 250 mg/kg/day rifaximin (Sigma-Aldrich Corp., USA, dissolved in corn oil), for 7 consecutive days, whereas the mice in the other groups were intragastically administered an equal amount of corn oil for 7 days. After 9 weeks, all mice were sacrificed through cervical dislocation and tissues were obtained.

### The evaluation of visceral sensitivity

Visceral sensitivity of NIH Swiss mice was measured by means of AWR tests to CRD, which has become an effective index to evaluate the changes of visceral sensitivity in mice because of its simple operation and intuitive results [24] CRD and AWR semi-quantitative score was carried out according to the method described by previous description [25]. Plastic balloon was inflated to 20, 40, 60 and 80 mmHg, AWR score and the threshold intensity were recorded at each value. The threshold of CRD was defined as the minimal distension volume evoked a visually evident contraction of the abdominal wall. Two observers recorded AWR grading and minimal distension separately and balloon inflation was done 3 times for each value to ensure the reliability of the results.

### Sample collection and DNA extraction

Isolation of fresh fecal pellets was done before mice were sacrificed via CO2 anesthetization followed by cervical dislocation. Then the colonic (3 cm proximal of the annus) and ileal (3 cm proximal of the ileocecal junction) tissues were harvested immediately. QIAamp DNA isolation kit (Qiagen, Valencia, CA, USA) was used to extract the total bacterial DNA. 16S RNA gene V4 region were amplified with the primers (Forward: 5’-AYTGGGYDTAAAGNG -3’; Reverse: 5’-TACNVGGGTATCTAATCC-3’), and then sequencing was performed on Illumina MiSeq platform (Illumina, San Diego, CA). Sequences were trimmed as described in a previous study21. Operational taxonomic units (OTUs) were clustered with a threshold of 97% similarity, and assigned taxonomically according to Silva database.

Bioinformatics analysis: Shannon index was used to evaluate diversity of microbiota in different groups. The general variation of the microbiota among the different groups were estimated by Principle coordinate analysis (PCoA) based on the the Unweighted Unifrac distances.

### Statistical analysis

AWR scores at each pressure of CRD and microbial data from 16S rRNA sequencing were compared among the three groups using the Kruskal–Wallis test, if the result was significant (*P* < 0.05), a Wilcoxon rank sum test with a Bonferroni correction at 0.05/3 was used to correct for multiple comparisons. The correlation between the relative abundance of microbiota different intestinal segments, of ileum mucosal microbiota and visceral sensation was analyzed using Pearson correlation test with p values corrected by FDR method. Other results are presented as mean ± SEM, and were compared by using one-way ANOVA, followed by the least significant difference (LSD) test or Dunnett's T3 test, for multiple comparisons as appropriate. A value of *P* < 0.05 was considered statistically significant. Statistical analysis was performed with SPSS version 19.

## Results

### Rifaximin could improve visceral hypersensitivity of PI-IBS mice

**Fig. 1:**
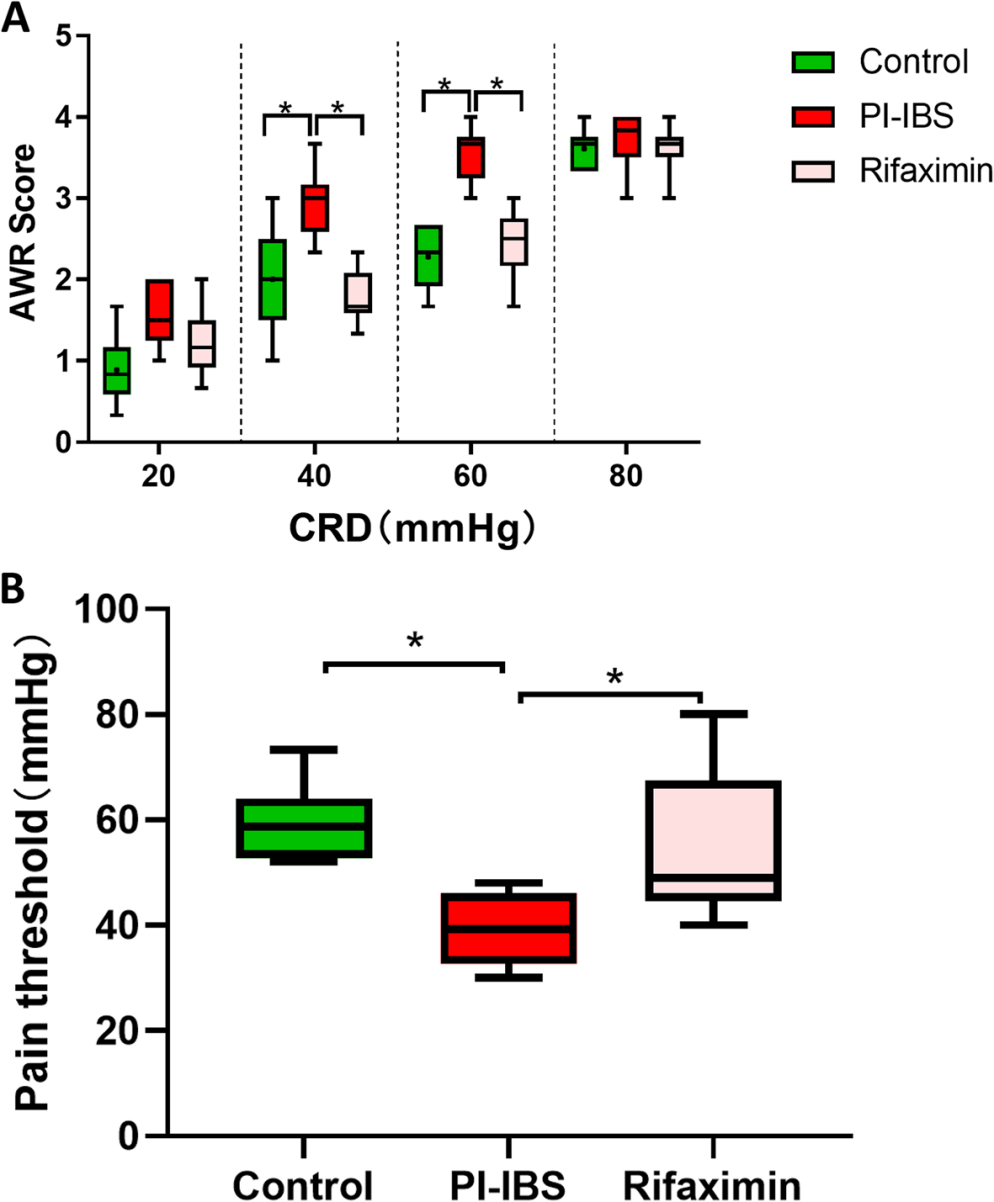
Effect of rifaximin on visceral sensation. **A** Box plot of AWR scores; **B** Pain thresholds of the CRD intensities. Boxes denote interquartile ranges and lines represent the median within the box. The whiskers define the 5th and 95th centiles, *n* = 8 mice per group. *p < 0.05

As shown in Fig. 1, compared with the control group, the AWR scores in the PI-IBS group was obviously increased under the colon inflating condition of 40 mmHg (*P* = 0.016, Fig. 1A) and 60 mmHg (*P* = 0.004, Fig. 1A) and the pain threshold was dramatically decreased (*P* = 0.032, Fig. 1B). These results indicated that a PI-IBS model with high visceral sensitivity was successfully established in *Trichinella spiralis*-infected mice. After rifaximin administration, the AWR scores of the treated mice were significantly decreased at both 40 mmHg (*P* = 0.005, Fig. 1A) and 60 mmHg (*P* = 0.043, Fig. 1A) compared with the PI-IBS group, meanwhile the pain threshold was increased (*P* = 0.032, Fig. 1B).

### Rifaximin affected the diversity of intestinal microbiota in different intestinal segments of PI-IBS mice

**Fig. 2:**
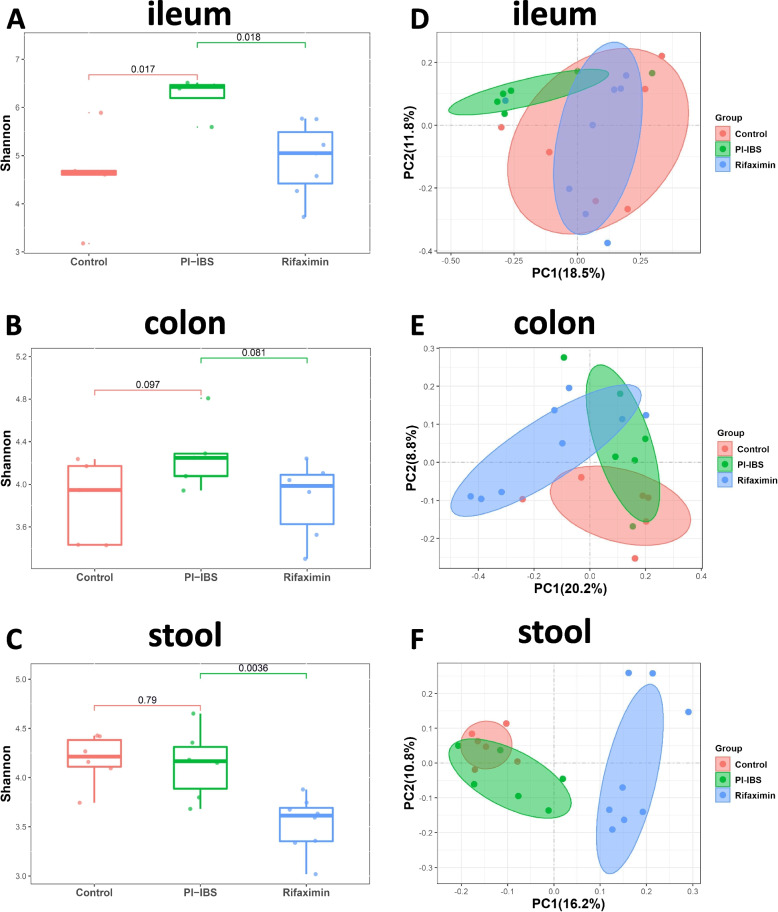
Comparisons of the alpha-diversity (**A-C**) and beta-diversity (**D-F**) between rifaximin treatment, PI-IBS and healthy controls of the samples from ileum, colon and stool. Boxes denote interquartile ranges and lines represent the median within the box. The whiskers represent maximum and minimum values respectively. Outliers are indicated by dots. *n* = 8 mice per group. *p < 0.05

In Fig. 2, we showed the diversity of intestinal microbiota in different intestinal segments, which was based on the Shannon index. We found that the diversity of the ileum mucosal microbiota in the PI-IBS group was significantly higher than that in the control group (*P* = 0.017, Fig. 2A). However, difference of microbial diversity between PI-IBS and control group in colonic mucosa (*P* = 0.097, Fig. 2B) and feces (*P* = 0.79, Fig. 2C) were not obvious. Interestingly, rifaximin treatment reduced the microbiota diversity in ileum mucosa (*P* = 0.018, Fig. 2A) and feces (*P* = 0.0036, Fig. 2C) of PI-IBS mice, whereas no significant changes were found in colon samples (*P* = 0.081, Fig. 2B). In order to evaluate the differences in microbial composition and diversity between samples, we performed principal coordinate analysis based on unweighted UniFrac distance. The results showed that compared with the control group, the ileum mucosal (Fig. 2D), colonic mucosal (Fig. 2E) and fecal microbiota (Fig. 2F) of the PI-IBS group all showed separate aggregation. Surprisingly, the ileum mucosa microbiota structure of the PI-IBS mice returned to a similar state to that of the control group after rifaximin treatment (Fig. 2D), while the colonic mucosa and fecal microbiota did not show such changes.

### Rifaximin had different effects on the composition of intestinal microbiota in different intestinal segments of PI-IBS mice

**Fig. 3:**
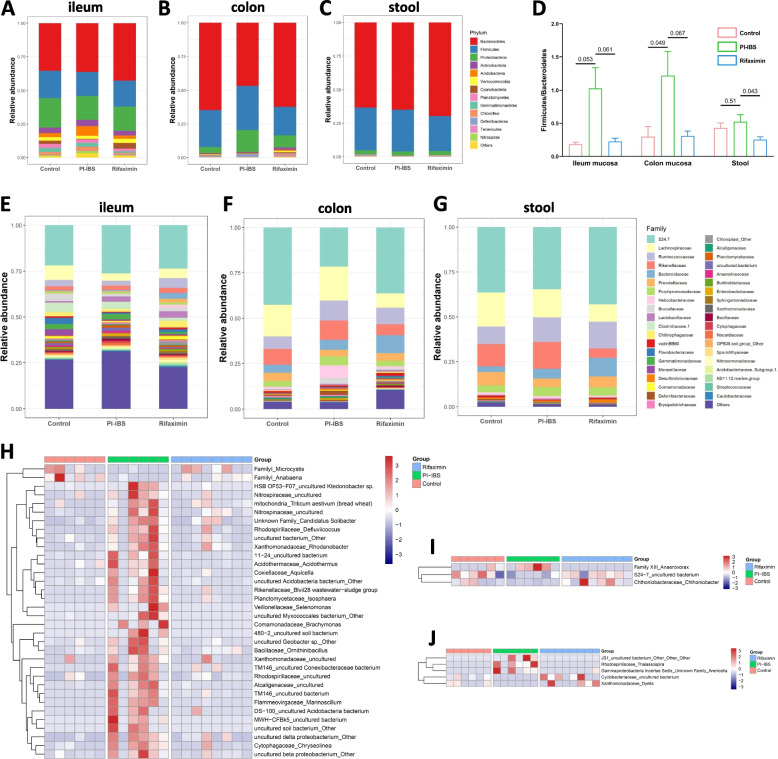
Variations of fecal microbiota composition between different groups. Relative proportions of bacterial phyla (**A-C**) and families (**E–G**) between three groups in ileum, colon and stool samples. **D** The ratio of Firmicutes to Bacteroidetes in colon and ileum of the three groups. Heatmap showing the abundances of bacterial genera in different groups from ileum (**H**), colon (**I**) and stool samples (**J**)

We analyzed the differences in the abundance of bacteria at three levels: phylum, family and genus. As shown in Fig. 3, at the phylum level, compared with the control group, the F/B(*Firmicutes/Bacteroidetes*) ratio of ileum mucosa (*P* = 0.053, Fig. 3D), colonic mucosa (*P* = 0.049, Fig. 3D) and feces (*P* = 0.51, Fig. 3D) increased in the PI-IBS group, and decreased after rifaximin treatment (ileum mucosa, *P* = 0.061; colonic mucosa, *P* = 0.067; feces, *P* = 0.043). In addition, we found that after rifaximin treatment, the relative abundance of *Cyanobacteria* in ileum mucosa increased, while *Acidobacteria*, *Chloroflexi* and *Nitrospirae* decreased (Fig. 3A). In colonic mucosa, the relative abundance of *Proteobateria* and *Deferribacteres* were decreased after rifaximin treatment (Fig. 3B). Interestingly, the relative abundance of fecal microbiota at the phylum level was not different among the three groups (Fig. 3C). At the family level, compared with PI-IBS group, *Lachnospiraceae*, *Brucellaceae*, and *Comamonadaceae* in ileum mucosa of PI-IBS mice increased after rifaximin treatment, while *S24.7* decreased (Fig. 3E). In colon mucosa, *S24.7* showed an increase, but the relative abundance of *lachnospiraceae*, *brucellaceae*, *helicobacteraceae* decreased after treatment with rifaximin (Fig. 3F). It was worth mentioning that differences in fecal microbiota were not obvious among the three groups (Fig. 3G).

Analysis of the heat map, we found 3 genera in colonic mucosa samples, 5 in feces but surprisingly, 34 species in ileal mucosa changed visibly after rifaximin treatment. Specifically, in ileum mucosa, the relative abundance of *Microcystis* and *Anabaena* decreased, *Triticum aestivum*, *Defluviicoccus* and other 32 genera showed an increase in PI-IBS group compared with the control (Fig. 3H). After treatment with rifaximin, the relative abundance of these genera returned to a similar level to that of the control group in ileum mucosa (Fig. 3H). In colon mucosa, rifaximin treatment decreased the relative abundance of *Anaerovorax*, and increased the relative abundance of *Chthoniobacter* in PI-IBS mice (Fig. 3I). Rifaximin treatment reversed the increase in the relative abundance of *Thalassospira* and *Arenicella* in the feces of the PI-IBS group, and obviously increased the relative abundance of *Dyella* (Fig. 3J).

### Rifaximin remodeled the microbiota association between different intestinal segments in IBS mice

**Fig. 4:**
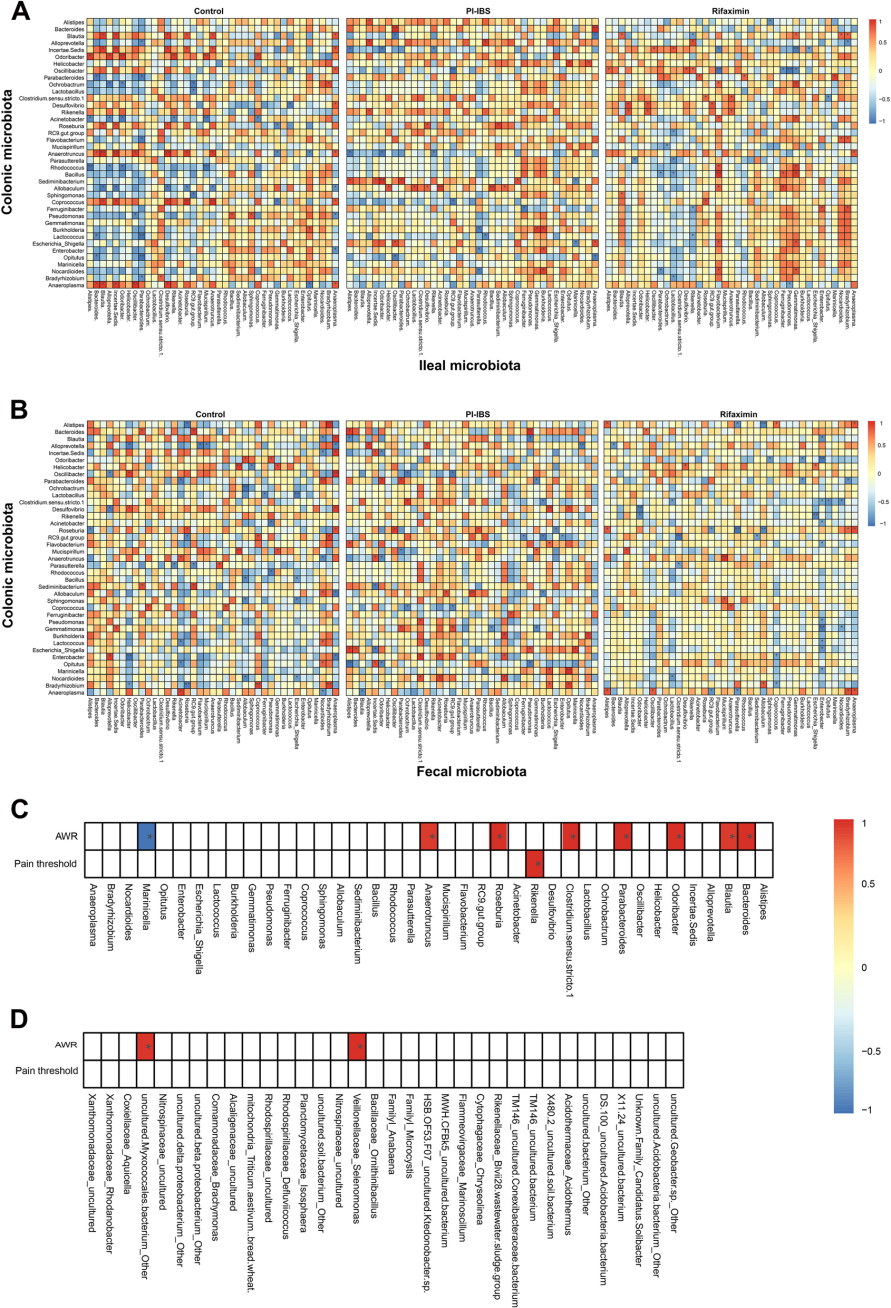
Correlation between ileal mucosal dominant genera and colonic mucosal dominant genera (**A**). Correlation between colonic dominant genera and fecal dominant genera (**B**). Correlation between ileal mucosal dominant genera (**C**), genera that showed significant changes after rifaximin intervention (**D** and visceral hypersensitivity.Red represented positive correlation and blue represented negative correlation. *p < 0.05, **p < 0.01

In order to investigate whether rifaximin could change the association between microbiota from different sites, we analyzed the correlation of dominant genera from ileal, colonic mucosa and feces. Since the ileal mucosa and feces were too far apart, we did not analyze the association between these two sites. As shown in the Fig. 4, we found 88 groups of dominant genera in the ileal mucosa and colonic mucosa showed significant correlations in the control group and only 46 groups in the IBS group, while the rifaximin intervention enhanced this correlation, with 65 groups of significant correlations(Fig. 4A). The effect of rifaximin on the correlation between colonic mucosal and fecal dominant genera was not as pronounced as between the colonic and ileal mucosa, with 37 significant correlations in the control group and 33 in the IBS group, however for the rifaximin group, the number was 22 (Fig. 4B). Further comparison showed that the correlations in the IBS group were different from the control group, and the rifaximin intervention could reshape these correlations to some extent. As shown in Table S1, there were 3 groups of dominant genera between colonic mucosa and ileal mucosa whose correlations were not significant in the IBS group and were strengthened after rifaximin treatment. The same situation was observed in the comparison of dominant genera between colonic mucosa and feces (Table S2). More generally, there was an emerging correlation between different intestinal segments of the genera in the IBS group compared with the control group, and this abnormal correlation was eliminated by the rifaximin treatment.

To further investigate the association between the alteration of intestinal microbiota by Rifaximin and the improvement of visceral hypersensitivity symptoms, we selected 39 dominant genera with the highest relative abundance in the ileal mucosa and 34 genera that showed significant changes after Rifaximin intervention. Then we explored their correlation with pain thresholds and AWR scores. We found a significant positive correlation between the relative abundance of *Rikenella* and pain thresholds (Fig. 4C). For AWR scores, in total, we found significant associations between AWR scores and 10 genera, eight of which were from ileal mucosal dominant genera(Fig. 4C) and two of which belonged to the genera with significant changes after the rifaximin intervention(Fig. 4D). Among these, all nine genera were positively correlated with the corresponding AWR scores, except for the *Marinicella*, which showed a significant negative correlation with the AWR scores. Interestingly, it was worth noting that *Bacteroides*, *Blautia*, *Odoribacter*, *Parabacteroides*, *Clostridium.*sensu*.stricto.1*, *Rikenella*, *Anaerotruncus* and *Marinicella* showed correlation with visceral hypersensitivity symptoms showed significant changes in their correlation with microbiota from other intestinal segments when compared with the control group, and these changes of correlation were completely corrected after rifaximin treatment.

## Discussion

In this study, a PI-IBS model was established after *Trichinella spiralis* infection. We found that rifaximin could significantly improve the visceral hypersensitivity in PI-IBS mice. Through the analysis of microbiota in ileum, colonic mucosa and feces, we found that rifaximin had different effects on microbiota from different intestinal segments, among which the effect of rifaximin on ileal mucosal microbiota was the most obvious. We found a correlation between ileal mucosal microbiota and visceral hypersensitivity symptoms of IBS. Rifaximin remodeled the ileal mucosal microbiota and the association of microbiota between different intestinal segments of PI-IBS to a composition similar to that of the control group, which might be one of the possible reasons for the improvement of IBS symptoms.

Visceral hypersensitivity, as a common pathophysiological condition of IBS, was mainly manifested as the decline of pain sensory threshold and closely related to the occurrence and development of IBS symptoms [26]. we observed that rifaximin could significantly reduce the AWR score and increase the pain threshold in PI-IBS mice. and it was worth noting that there was a close relationship between intestinal microbiota and the development of visceral hypersensitivity [8, 26]. Previous studies on rifaximin usually used feces as the primary source of samples, which ignored the fact that there were differences between mucosa and fecal microbiota. Fecal microbes could not be used as a substitute for microbes in other parts of intestine [27, 28].In addition, because of rifaximin’s different drug utilization rates and different microbial composition in different intestinal parts, it was reasonable to assume that rifaximin had different effects on microbiota in different intestinal segments.

We found that diversity of bacteria in ileum mucosa of PI-IBS increased, while the diversity in colonic mucosa and fecal samples did not increase significantly. In this model, the alpha diversity of ileal mucosal microbiota of PI-IBS increased, while in another study, the diversity of ileal mucosal microbiota decreased in the IBS group [16]. It was worth noting that this IBS model was induced by chronic water withdrawal or repeated restraint stress, rather than an infection-associated IBS model. Whether this difference in modeling method was the reason for the completely opposite characteristics of ileal mucosa microbiota required further research. In this experiment, rifaximin mainly worked in the small intestine, and owing to its broad-spectrum antibacterial effect, it reduced the diversity of ileal mucosal microbiota. However, due to the low drug availability of rifaximin in colon, the change of colonic mucosal microbiota diversity was not obvious. Rifaximin also showed obvious inhibitory effect on the diversity of fecal microbiota because fecal microbiota could come from various parts of the intestinal, which might include the sites where rifaximin is active. Similar to the results of our experiment, a clinical trial study for patients with diarrhoea-predominant IBS also found the Shannon diversity and richness of fecal microbiota slightly decreased after rifaximin treatment [29]. It is worth noting that in this study the change in microbiota diversity is short-term. As for how long rifaximin can maintain the change in microbiota diversity, and whether the duration of influence between different intestinal segments is different, this may require more in-depth research to clarify.

By analyzing the inter-sample diversity (β diversity), we found that rifaximin restored a highly similar bacterial structure to that of the control group in the ileal mucosa of PI-IBS mice. Previous studies had suggested that changes in the microbial composition of the small intestine might be one of underlying mechanisms of IBS [30]. Therefore, rifaximin improving ileal microbial dysbiosis in PI-IBS mice might be helpful to relieve the symptoms of IBS. In the colon and stool samples, the newly formed microbial colony after rifaximin treatment in PI-IBS mice showed completely different composition characteristics from that of the control, which also suggested that the effect of rifaximin on intestinal microbiota of PI-IBS mice was different in different intestinal segments. The effect of rifaximin on the improvement of IBS might mainly be reflected in the ileum, and the regulation of colonic mucosal microbiota might not have much to do with the improvement of IBS symptoms.

We analyzed different parts of the microbiota at phylum, family and genus level again and found that rifaximin could change the relative abundance of microbiota in different intestinal segments of PI-IBS mice. At the phylum level, we have observed the change of F/B ratio. There was an increase in the F/B ratio of PI-IBS mice, which was evident in ileum, colonic mucosa and feces and could be reduced by rifaximin. The change of F/B ratio was generally regarded as a sign of gut dysbiosis, and its increase might be associated with increased intestinal mucosal permeability and mild inflammation [31]. Previous studies had confirmed that gut dysbiosis and slight intestinal inflammatory activity could alter visceral sensation [32]. The decrease of F/B ratio in PI-IBS mice after rifaximin treatment might indicate that the disturbance of microbiota has been improved and intestinal inflammation has been alleviated, which might be one of the reasons for improving visceral hypersensitivity. At the family level, we found that the abundance of *Lachnospiraceae* in ileal mucosa decreased in PI-IBS mice and increased after treatment with rifaximin. Previous studies had suggested that the degree of depression in patients with IBS was negatively correlated with the relative abundance of *Lachnospiraceae* [33]. Another study also confirmed that the relative abundance of *Lachnospiraceae* decreased after GHT (Gut-directed hypnotherapy) [34]. The risk of anxiety and depression in patients with IBS was three times higher than that in normal people [35], and their abnormal brain function and pathological changes had also been confirmed [36]. The effect of rifaximin on the relative abundance of *Lachnospiraceae* in PI-IBS mice might reflect its regulatory effect on brain and intestinal dysfunction, which might also be one of the reasons for reducing visceral hypersensitivity. It was worth noting that after rifaximin treatment, the change of relative abundance of *Lachnospiraceae* in colonic mucosa and feces was not similar to that in ileal mucosa, which might be due to the difference in antimicrobial effect of rifaximin in different intestinal segments. Studies have shown that the solubility of rifaximin in the bile environment of the small intestine is increased 70–120 fold [37], which helps to reduces its particle size and facilitates its entrance into the bacterial cell. Another study simulating the small intestinal fluid environment in vitro also proved that rifaximin had a stronger antimicrobial effect on *E. coli*, *Kleb-siella spp.*, *Enterobacter spp.* and *E. faecalis* that are the main SIBO pathogens in solutions containing bile acids [38]. These results once again proved that the improvement effect of rifaximin on IBS might be closely related to different intestinal sites.

We analyzed the microbiota that had apparent differences at genus level among groups (*P* < 0.05). We found that the difference of ileal mucosa microbiota was the most obvious. Compared with the control group, the relative abundance of thirty-four different genera in the PI-IBS group changed in ileal mucosa, and only three and five genera changed in the colonic mucosa and feces, respectively. This difference further proved that PI-IBS had the most significant effect on the composition of ileal microbiota in mice. Interestingly, compared with the control group, most of the changes in the ileal microbiota of PI-IBS mice were an increase in the relative abundance of microbiota. Whether this change had physiological significance needs to be confirmed by more in-depth studies.

Changes in the relative abundance of the microbiota were associated with interactions between different bacteria. Studies had found that alterations in the composition and/or activity of the microbiota and bacterial metabolites such as short-chain fatty acids (SCFAs) could activate the host immune system, promote cytokine production and affect intestinal physical and chemical environment, which could affect the colonization of microbiota in the intestinal tract [39]. While it was not clear whether there were interactions between microbiota from different intestinal segments which were spatially distant exist. Our analysis found that rifaximin reshaped the correlation changed by IBS between ileal and colonic mucosa, whereas the effects were not evident when comparing colonic mucosal and fecal microbiota. In this study, we further demonstrated a positive correlation between ileal mucosal microbiota and AWR scores, suggesting that these genera might be potential detriments factors for visceral hypersensitivity. Among them,the relative abundance of *Bacteroides* [39, 40] , *Blautia* [41] , *Odoribacter* [5] , *Parabacteroides* [40] , *Clostridum.*sensu*.stricto.1* [7] increased in IBS patients. Interestingly, we also found a negative correlation between the relative abundance of *Marinicella* and the AWR score, suggesting that intestinal microbiota might also be protective factors for visceral hypersensitivity. It was worth noting that these genera that showed correlation with IBS symptoms showed significant changes in their correlation with microbiota from other intestinal segments when compared with the control group and these changes of correlation were completely corrected after rifaximin treatment. These result provides a possible hypothesis that the microbiota in different intestinal segments might influence each other. Rifaximin was able to change the structure of colonic microbiota by affecting the abundance of related bacteria in the ileum, which further leads to the occurrence of IBS symptoms. Whether these abnormal correlations were related to the appearance of IBS symptoms and whether its correction by rifaximin was one of the mechanisms of its therapeutic action require more in-depth studies.

There were also some limitations in the study. We did not analyze and predict the changes of microbiota function that may occur with the change of microbiota structure. In terms of experimental design, due to the significant differences between groups, we did not design a pre-and post-control to evaluate the impact of interventions within the group. And our study did not analyze the total amount of bacteria in the small intestine before and after rifaximin intervention, which made it impossible to judge the existence of SIBO and the possible effect of rifaximin on it.

In conclusion, rifaximin could dramatically improve visceral hypersensitivity in PI-IBS mice. Rifaximin mainly affects ileal mucosal microbiota, and its improvement effect on IBS might be closely related to the improvement of ileal microbiota structure.

## Supplementary Information

**Additional file 1: Table S1.** Correlation analysis of dominant bacterial genera between colonic and ileal mucosa. **Table S2.** Correlation analysis of dominant bacterial genera between colonic mucosa and feces.

## Data Availability

The RNA sequences (accession number: CRA007438) are available in Genome Sequence Archive at the BIG Submission (BIG Sub: https://ngdc.cncb.ac.cn/gsub/).The datasets used and analyzed during the current study are available from the corresponding author on reasonable request.

## Abbreviations

IBS-D: Diarrhea-predominant irritable bowel syndrome
SIBO: Small intestinal bacterial overgrowth
HC: Healthy control
PI-IBS: Post-infectious irritable bowel syndrome
AWR: Abdominal withdrawal reflex
