# Supplementary material for: Modulation of the microbiota across different intestinal segments by Rifaximin in PI-IBS mice
Source: BMC Microbiol. 2023 Jan 19;23:22. doi: 10.1186/s12866-023-02772-6 (PMC9850553; doi:10.1186/s12866-023-02772-6)
Supplement: Supplementary file 1 — Additional file 1: Table S1. Correlation analysis of dominant bacterial genera between colonic and ileal mucosa. Table S2. Correlation analysis of dominant bacterial genera between colonic mucosa and feces. [file 12866_2023_2772_MOESM1_ESM.docx]

**Table.S1 Correlation analysis of dominant bacterial genera between colonic and ileal mucosa**

| Group | | Health Control | | | IBS Rifaximin | | | |
| --- | --- | --- | --- | --- | --- | --- | --- | --- |
|  |  | **R** | **P** | **R** | | **P** | **R** | **P** |
| Colon Ileum | |  |  |  | |  |  |  |
| Blautia Blautia | | 0.94 | 0.02 | 0.09 | | 0.92 | 0.74 | 0.05 |
| Coprococcus Roseburia | | 1.00 | 0.00 | -0.49 | | 0.36 | 0.81 | 0.02 |
| Anaerotruncus Anaeroplasma | | 0.85 | 0.03 | -0.77 | | 0.10 | 0.76 | 0.04 |
| Incertae.Sedis Alistipes | | 0.26 | 0.66 | -0.94 | | 0.02 | 0.43 | 0.30 |
| Anaerotruncus Alistipes | | 0.49 | 0.36 | -0.94 | | 0.02 | 0.64 | 0.10 |
| Incertae.Sedis Odoribacter | | 0.37 | 0.50 | -0.94 | | 0.02 | 0.10 | 0.84 |
| Anaerotruncus Odoribacter | | 0.60 | 0.24 | -0.94 | | 0.02 | 0.52 | 0.20 |
| Incertae.Sedis Rikenella | | 0.60 | 0.24 | -0.89 | | 0.03 | 0.51 | 0.19 |
| Anaerotruncus Rikenella | | 0.77 | 0.10 | -0.89 | | 0.03 | 0.41 | 0.31 |
| Oscillibacter RC9.gut.group | | 0.03 | 1.00 | -1.00 | | 0.00 | 0.62 | 0.11 |
| Allobaculum Bacillus | | 0.43 | 0.42 | 0.94 | | 0.01 | 0.17 | 0.69 |
| Allobaculum Allobaculum | | 0.37 | 0.50 | 0.82 | | 0.05 | 0.08 | 0.84 |
| Ferruginibacter Ferruginibacter | | 0.14 | 0.80 | -0.94 | | 0.02 | 0.60 | 0.13 |
| Burkholderia Gemmatimonas | | 0.54 | 0.30 | 0.94 | | 0.02 | 0.45 | 0.27 |
| Nocardioides Burkholderia | | 0.31 | 0.56 | 0.94 | | 0.02 | 0.19 | 0.66 |
| Helicobacter Bradyrhizobium | | 0.83 | 0.06 | 0.89 | | 0.03 | 0.26 | 0.54 |
| Sediminibacterium Alistipes | | -0.03 | 1.00 | 0.89 | | 0.03 | -0.05 | 0.93 |
| Allobaculum Helicobacter | | -0.49 | 0.36 | 0.88 | | 0.02 | -0.06 | 0.89 |
| Bacteroides Oscillibacter | | -0.60 | 0.24 | 1.00 | | 0.00 | -0.29 | 0.50 |
| Escherichia_Shigella Oscillibacter | | -0.20 | 0.71 | 0.89 | | 0.03 | -0.55 | 0.17 |
| Sediminibacterium Parabacteroides | | -0.77 | 0.10 | 0.94 | | 0.02 | -0.21 | 0.62 |
| Burkholderia Parasutterella | | -0.37 | 0.50 | -0.94 | | 0.02 | -0.14 | 0.75 |
| Nocardioides Parasutterella | | -0.14 | 0.80 | -0.94 | | 0.02 | -0.36 | 0.39 |
| Alloprevotella Rhodococcus | | -0.03 | 1.00 | 0.94 | | 0.02 | -0.24 | 0.58 |
| Lactococcus Rhodococcus | | -0.14 | 0.80 | -0.89 | | 0.03 | -0.26 | 0.54 |
| Roseburia Sediminibacterium | | -0.43 | 0.42 | 0.94 | | 0.02 | -0.36 | 0.39 |
| Bacteroides Anaeroplasma | | -0.14 | 0.80 | 1.00 | | 0.00 | -0.71 | 0.06 |

**Table.S2 Correlation analysis of dominant bacterial genera between colonic mucosa and feces**

| Group | | Health Control | | | IBS Rifaximin | | | |
| --- | --- | --- | --- | --- | --- | --- | --- | --- |
|  |  | **R** | **P** | **R** | | **P** | **R** | **P** |
| Colon Feces | |  |  |  | |  |  |  |
| Roseburia Anaeroplasma | | 0.89 | 0.03 | -0.43 | | 0.42 | 0.83 | 0.02 |
| Alloprevotella Alloprevotella | | 0.83 | 0.06 | 0.94 | | 0.02 | 0.62 | 0.11 |
| RC9.gut.group Burkholderia | | 0.03 | 1.00 | -0.94 | | 0.02 | 0.31 | 0.46 |
| Allobaculum Burkholderia | | 0.14 | 0.80 | -0.88 | | 0.02 | 0.04 | 0.93 |
| RC9.gut.group Enterobacter | | 0.65 | 0.16 | -0.82 | | 0.05 | 0.12 | 0.77 |
| Incertae.Sedis Incertae.Sedis | | 0.71 | 0.14 | 0.89 | | 0.03 | 0.21 | 0.62 |
| Anaerotruncus Incertae.Sedis | | 0.60 | 0.24 | 0.89 | | 0.03 | 0.17 | 0.70 |
| Sediminibacterium Incertae.Sedis | | 0.37 | 0.50 | -0.94 | | 0.02 | 0.33 | 0.43 |
| Opitutus Incertae.Sedis | | 0.09 | 0.92 | 0.89 | | 0.03 | 0.24 | 0.57 |
| Desulfovibrio Lactococcus | | 0.26 | 0.66 | -0.94 | | 0.01 | 0.05 | 0.93 |
| Flavobacterium Lactococcus | | 0.09 | 0.92 | 0.81 | | 0.05 | 0.19 | 0.66 |
| Alloprevotella Nocardioides | | 0.75 | 0.08 | -0.93 | | 0.01 | 0.24 | 0.56 |
| Oscillibacter Ochrobactrum | | 0.49 | 0.36 | -0.94 | | 0.02 | 0.55 | 0.17 |
| Roseburia Oscillibacter | | 0.26 | 0.66 | 0.94 | | 0.02 | 0.43 | 0.30 |
| Parabacteroides Parabacteroides | | 0.54 | 0.30 | 0.94 | | 0.02 | 0.64 | 0.10 |
| Mucispirillum Parabacteroides | | 0.09 | 0.92 | -0.89 | | 0.03 | 0.12 | 0.79 |
| Gemmatimonas Parabacteroides | | 0.58 | 0.23 | 0.89 | | 0.03 | 0.52 | 0.20 |
| Bacteroides Pseudomonas | | 0.26 | 0.66 | 1.00 | | 0.00 | 0.29 | 0.50 |
| Opitutus Pseudomonas | | 0.14 | 0.80 | -0.94 | | 0.02 | 0.47 | 0.24 |
| Gemmatimonas RC9.gut.group | | 0.67 | 0.15 | 0.89 | | 0.03 | 0.19 | 0.66 |
| Escherichia_Shigella Alistipes | | -0.37 | 0.50 | 0.94 | | 0.02 | -0.02 | 0.98 |
| Allobaculum Anaerotruncus | | -0.49 | 0.36 | 0.94 | | 0.01 | -0.37 | 0.37 |
| Anaeroplasma Blautia | | -0.09 | 0.92 | -0.89 | | 0.03 | -0.14 | 0.75 |
| Clostridium.sensu.stricto.1 Burkholderia | | -0.43 | 0.42 | -0.89 | | 0.03 | -0.33 | 0.43 |
| Pseudomonas Clostridium.sensu.stricto.1 | | -0.31 | 0.56 | 0.89 | | 0.03 | -0.24 | 0.56 |
| Enterobacter Clostridium.sensu.stricto.1 | | -0.37 | 0.50 | 0.94 | | 0.02 | -0.12 | 0.77 |
| Nocardioides Clostridium.sensu.stricto.1 | | -0.26 | 0.66 | 0.89 | | 0.03 | -0.32 | 0.44 |
| Roseburia Escherichia_Shigella | | -0.03 | 0.95 | -0.89 | | 0.03 | -0.08 | 0.84 |
| RC9.gut.group Ferruginibacter | | -0.14 | 0.80 | -0.87 | | 0.02 | -0.17 | 0.70 |
| Parabacteroides Gemmatimonas | | -0.03 | 0.95 | -0.88 | | 0.02 | -0.07 | 0.88 |
| Gemmatimonas Gemmatimonas | | -0.06 | 0.91 | -0.88 | | 0.02 | -0.07 | 0.88 |
| Escherichia_Shigella Marinicella | | -0.37 | 0.50 | 0.94 | | 0.02 | -0.57 | 0.15 |
| Parabacteroides Odoribacter | | -0.14 | 0.80 | -0.89 | | 0.02 | -0.10 | 0.84 |
| Bradyrhizobium Opitutus | | -0.03 | 0.96 | 0.94 | | 0.02 | -0.19 | 0.66 |
| Escherichia_Shigella Pseudomonas | | -0.54 | 0.30 | 0.89 | | 0.03 | -0.40 | 0.33 |
| Oscillibacter RC9.gut.group | | -0.03 | 1.00 | -0.94 | | 0.02 | -0.05 | 0.93 |
| Coprococcus RC9.gut.group | | -0.14 | 0.80 | -0.89 | | 0.03 | -0.10 | 0.84 |
| Ferruginibacter Sphingomonas | | -0.31 | 0.56 | 0.89 | | 0.03 | -0.14 | 0.75 |
